# Prediction of immune infiltration and prognosis for patients with urothelial bladder cancer based on the DNA damage repair-related genes signature

**DOI:** 10.1016/j.heliyon.2023.e13661

**Published:** 2023-02-13

**Authors:** Tianhang Li, Ning Jiang, Yuhao Bai, Tianyao Liu, Zihan Zhao, Xinyan Xu, Yulin Zhang, Fayun Wei, Rui Sun, Siyang Liu, Jiazheng Li, Hongqian Guo, Rong Yang

**Affiliations:** a Department of Urology, Affiliated Nanjing Drum Tower Hospital, Medical School, Nanjing University, Nanjing, China; b Nanjing Drum Tower Hospital Clinical College of Jiangsu University, Nanjing, China; c Nanjing Drum Tower Hospital Clinical College of Nanjing University of Chinese Medicine, Nanjing, China

**Keywords:** Bladder cancer, Prognosis, DNA Damage repair, Immunotherapy

## Abstract

**Objectives:**

To analyze the correlations between the expression and effect of DNA damage repair genes and the immune status and clinical outcomes of urothelial bladder cancer (BLCA) patients. In addition, we evaluate the efficacy and value of utilizing the DNA damage repair genes signature as a prognosis model for BLCA.

**Methods:**

Two subtype groups (C1 and C2) were produced based on the varied expression of DNA damage repair genes. Significantly differentiated genes and predicted enriched gene pathways were obtained between the two subtypes. Seven key genes were obtained from the DNA damage repair-related genes and a 7-gene signature prognosis model was established based on the key genes. The efficacy and accuracy of this model in prognosis prediction was evaluated and verified in two independent databases. Also, the difference in biological functions, drug sensitivity, immune infiltration and affinity between the high-risk group and low-risk group was analyzed.

**Results:**

The DNA damage repair gene signature could significantly differentiate the BLCA into two molecular subgroups with varied genetic expression and enriched gene pathways. Seven key genes were screened out from the 232 candidate genes for prognosis prediction and a 7-gene signature prognosis model was established based on them. Two independent patient cohorts (TCGA cohort and GEO cohort) were utilized to validate the efficacy of the prognosis model, which demonstrated an effective capability to differentiate and predict the overall survival of BLCA patients. Also, the high-risk group and low-risk group derived from the 7-gene model exhibited significantly differences in drug sensitivity, immune infiltration status and biological pathways enrichment.

**Conclusions:**

Our established 7-gene signature model based on the DNA damage repair genes could serve as a novel prognosis predictive tool for BLCA. The differentiation of BLCA patients based on the 7-gene signature model may be of great value for the appropriate selection of specific chemotherapy agents and immune-checkpoint blockade therapy administration.

## 1 Introduction

Bladder cancer (BLCA) is one of the most common urological malignancies globally, leading in both incidence rate and mortality rate [1,2]. Smoking has been identified as a major contributing factor for the oncogenesis of BLCA [3]. Clinically, BLCA could be divided into two subtypes as non-muscle invasive bladder cancer (NMIBC) and muscle-invasive bladder cancer (MIBC), which is based on the degree of tumor penetration into the muscle layer. By timely inhibitory therapeutics such as transurethral resection of bladder tumor (TURBT) and intravesical Bacille Calmette-Guérin (BCG) infusion treatment, A nearly 90% 5-year survival rate could be achieved in the NMIBC patients. However, what we cannot neglect is that NMIBC still has a high tendency of recurrence and progression into MIBC [4,5]. Once BLCA patients progressed into MIBC, the 5-year rate would drastically decline into 50% and the probability of further metastasis would sharply increase [6,7]. Radical cystectomy in combination with neoadjuvant chemotherapy has long been utilized as the standard therapeutic for MIBC, while the high operation difficulty, multiple complications as well as the expensive surgical cost largely limited the patient benefits [8,9]. Recently, immunotherapy based on immune-checkpoint blockade (ICB) has shed a new light of hope on the clinical treatment of cancer, whose efficacy has been verified in a range of clinical trials [[10], [11], [12]]. Nevertheless, a great efficacy difference was observed among individuals, which suggested that the genetic and immune characteristics may determine the immune sensitivity of the BLCA patients and more detailed patient selection and grouping strategies based on gene signature are urgently warranted [13]. In addition, innate or adaptive immune resistance seriously dampened the effectiveness of ICB, which indicated that a deeper understanding of regulatory mechanisms involving antitumor immunity is of great importance [13,14].

A good preservation of the genomic information is an indispensable premise for the stability and homeostasis of an independent life. Meanwhile, genetic instability, mainly represented by mutagenesis, is the driving force for the evolution of life. The balance between these two divergent pathways ensured the perpetuation of life, and DNA damage repair (DDR) pathway is an integral part of this network [15,16]. Owing to high susceptibility to both exogenous and endogenous inducing factors, as well as the inevitable incidence of mistaken replication or repair of DNA, unfavorable mutations could emerge and accumulate in cells. Thanks to the DDR system, the damage to DNA could be eliminated timely and thereby avoiding disadvantageous mutations [16,17]. However, inappropriate regulation or functional defects of DDR system could be hijacked by tumor cells, which may help promote the formation of various hallmarks of cancer [18,19]. Recently, multiple studies have found that DDR-related genes play pivotal roles in the process of a range of cancers [[20], [21], [22]]. Moreover, the DDR-related gene signature has been identified as a potential tool to predict the ICB efficacy [23]. In BLCA, DDR gene mutational status have been found to be associated with immune genes expression [24]. In addition, disturbance or activation of DDR pathways have been found to be promising therapeutic targets for multiple treatments, including radiation [25,26] and ICB immunotherapy [27]. Yin et al. made a primary evaluation of the prognostic value of DDR-related genomic alterations for BLCA patients and found that ATM alteration has a significant association with a worse prognosis [28]. However, sufficient data illustrating the prognostic value of DDR-related genes in BLCA and studies exploring the relationship between DDR and immune status of BLCA still lacked.

Here in this study, we established a prognosis model based on the expression of DDR-related genes in BLCA and made a deep analysis into the correlation between DDR and immune infiltration and tumor stemness. We found that the prognosis of BLCA patients could be effectively stratified and predicted. DDR pathway may provide more valuable information for the clinical diagnosis and treatment of BLCA.

## 2 Materials and methods

### 2.1 Data extraction and processing

The Cancer Genome Atlas (TCGA) database (https://portal.gdc.cancer.gov/) was utilized to collect the raw data of the RNA sequence-based counts along with the corresponding clinical parameters. A total of 408 BLCA patients and 19 normal tissues were finally enrolled in the cohort. For validation of our prognosis model, another testing cohort was extracted from the GENE EXPRESSION OMNIBUS (GEO) database. A total of 256 BLCA patients were finally enrolled from GSE13507 and GSE31684 datasets. The transcriptome information and the clinical parameters, including age, grade, TNM stage, and overall survival (OS) data were obtained. The data is shared voluntarily and could be downloaded and used freely in no requirement of ethics committee approval.

### 2.2 Molecular subgrouping method

To establish the molecular subtypes of BLCA based on DDR-related genes, the RNA-sequencing expression data (level 3) and corresponding clinical parameters were extracted. ConsensusClusterPlus R package (v1.54.0) was utilized to conduct the consistency analysis, the maximum number of clusters is 6, and 80% of the total sample is drawn 100 times, clusterAlg = “hc”, innerLinkage = ‘ward.D2’. The R software package pheatmap (v1.0.12) was used to draw the clustering heatmaps. The genes displayed in the heatmap are kept with SD > 0.1. Top 1/4 genes will be filtrated out depending on the SD if the input genes outnumbered 1000. Two subtypes (C1 and C2) were formed as shown in the heatmap. The list of all the DDR-related genes used in the analysis has been provided in the supplemental data of Table S1.

To further explore the genetic and molecular difference between C1 and C2 subtypes, the R limma package was used to obtain the differentially expressed mRNA. The threshold was set as “Log2F > 1 or Log2F < −1 with the adjusted p value < 0.05” for the differential expression of mRNAs. To further predict the biological functions of the DDR-derived molecular subtypes, functional pathway enrichment analysis was performed on the obtained significantly up- or down-regulated genes. R ClusterProfiler package was utilized to analyze the GO function prediction and the KEGG pathway enrichment. The boxplot and heatmap were drew via R ggplot2 package and pheatmap package respectively. All the analysis methods and R package were implemented by R version 4.0.3.

### 2.3 Establishment and assessment of the prognosis model

We first utilized the univariate Cox regression analysis to identify the crucial prognosis-associated genes (p < 0.01)**.** Ten key genes were selected from the significantly-associated genes infiltrated by univariate Cox regression analysis. The After screening out the 10 key genes. The R glmnet package was used to conduct the feature selection through the method of Least absolute shrinkage and selection operator (LASSO) regression algorithm. Survival outcomes were compared between groups by Log-rank testing KM survival analysis. The predictive efficacy and the risk score of DDR-related 7 key genes were evaluated by TimeROC analysis. Univariate Cox proportional hazards regression and log-rank test were utilized to calculate the p-values and hazard ratio (HR) with 95% confidence interval (CI) and generate the Kaplan–Meier curves. P < 0.05 was viewed as statistically significant for certain difference. The R forest package was used to draw the forest map, which showed the HR, 95% CI and the p value, by the univariate and multivariable Cox regression analysis. Based on the multivariate COX proportional hazards analysis results, we utilized the R RMS package to generate the nomogram to make a prediction of the 1, 3, 5-year recurrence rate.

### 2.4 Immune infiltration and GSEA gene pathways enrichment analysis

To explore the relationship between immune infiltration status and different DDR-divided subtypes in BLCA, we used the R package of immundeconv, which consists of six algorithms, including TIMER, xCell, MCP-counter, CIBERSORT, EPIC and quanTIseq. Each algorithm owns its specific advantages and unique characteristics. The immune function difference was compared between high-risk and low-risk groups through GSVA R package. Crucial small-molecular bioactive components between high-risk and low-risk groups of BLCA patients were compared through limma algorithm. To compare the expression distribution of immune checkpoints gene in high-risk and low-risk groups. RNA-sequencing expression (level 3) profiles and corresponding clinical information for BLCA were downloaded from the TCGA dataset (https://portal.gdc.com). A range of key immune-checkpoint proteins were selected and the expression levels of these genes were obtained. The ggplot2 R package and pheatmap R package were used for the analysis. Potential ICB response and immune dysfunction and exclusion status were predicted with TIDE algorithm. TCIA database was used to compare the T cell exhaustion status between high-risk and low-risk group through wilcox test.

Gene Set Enrichment Analysis (GSEA) (http://www.broad.mit.edu/gsea/) was utilized to determine the concordant and significant differences in the defined genes set and pathways between 2 biological conditions.

### 2.5 Drug sensitivity prediction analysis

The RNA-sequencing expression information and related clinical information were downloaded from the TCGA dataset. The chemotherapeutic response for each group was predicted based on the available pharmacogenomics database of the Genomics of Drug Sensitivity in Cancer (GDSC) (https://www.cancerrxgene.org). The R pRRophetic package was used to perform the prediction. The samples' half-maximal inhibitory concentration (IC50) was measured using ridge regression. All parameters were set as the default values. The batch effect of combat and tissue type were used for all tissues, and the duplicate gene expression was set as mean value.

## 3 Results

### 3.1 DDR-based molecular subtype grouping

**Fig. 1:**
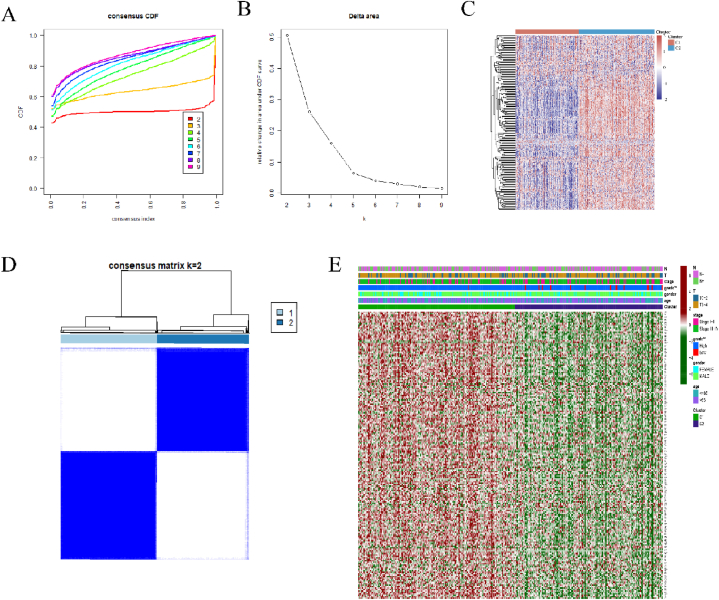
Molecular subtypes of BLCA based DDR-related genes. (A, B) Delta area curve of consensus clustering, representing the alterations in area under the cumulative distribution function (CDF) curve for each category number k compared with k − 1. (C) The heatmap showing differentiation of DDR-related gene expression in C1 and C2, the color of red and blue represents high and low expression respectively. (D) The heatmap illustrating consensus clustering solution (k = 2) for DDR-related genes. (E) Comparative analysis on the core clinical parameters. (For interpretation of the references to color in this figure legend, the reader is referred to the Web version of this article.)

**Fig. 2:**
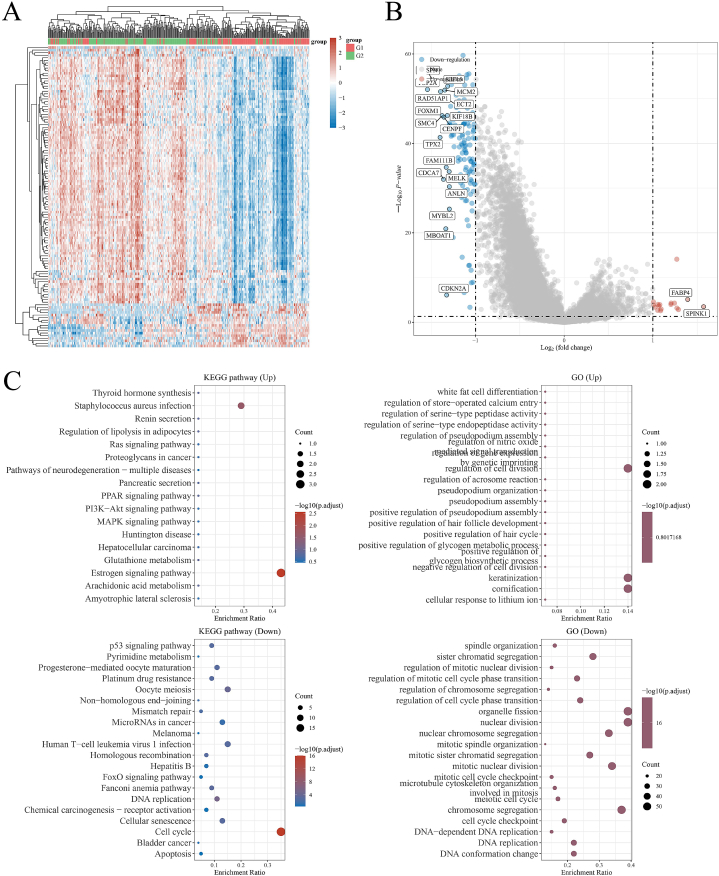
Genetic difference between the two molecular subtypes. (A) Hierarchical clustering analysis performed on the differentially-expressed mRNAs between the two groups. (B) Volcano plot showing differentially expressed genes between C1 and C2. (C) GO and KEGG analysis on the molecular subgroups.

**Fig. 3:**
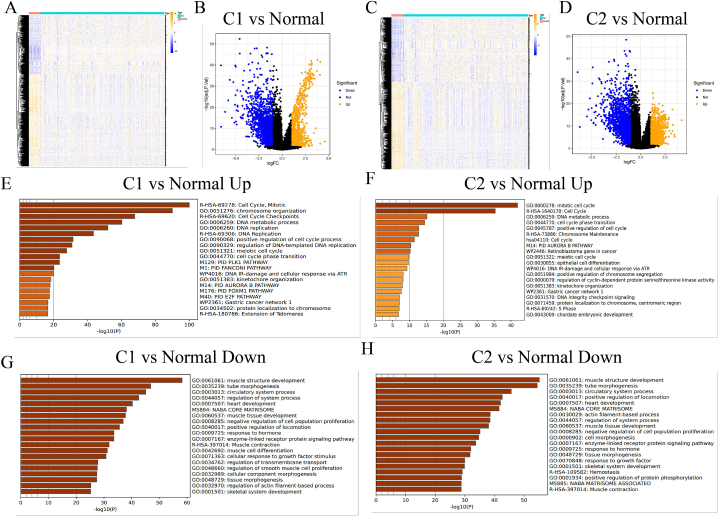
Genetic difference between C1, C2 and the uninvolved normal tissues. (A, C) Hierarchical clustering analysis performed on the differentially-expressed mRNAs between the two groups. (B, D) Volcano plot showing differentially expressed genes between C1 and C2. (E–H) Gene enrichment analysis on the C1, C2 and the normal tissues.

Distinct subgroups of 408 BLCA patients were generated according to the expression levels of DDR-related genes. Sufficient selection could be achieved when k was set as 2 (Fig. 1A–B), and two molecular subgroups were finally divided from the whole cohort. Two subgroups as C1 and C2 were successfully formed according to proportion of ambiguous clustering (PAC) (Fig. 1C–D). Comparative analysis on the core clinical parameters, including T and N stage, Tumor grade, gender, and age, was conducted between C1 and C2 (Fig. 1E). Significant difference was only identified in the tumor grade between C1 and C2. More low-grade patients were found in the C2 group. When it comes to tumor stage, gender, and age, no significant difference was found between the two groups. Differentially expressed genes (DEGs) were compared between the two subgroups. In addition, GO annotation and KEGG pathway enrichment analysis were performed to explore the downstream regulatory genetic functions (Fig. 2A–C). Also, a comparative analysis between C1 and C2 with the uninvolved normal tissues may also provide with more valuable and accurate information. Therefore, we also conducted a DEGs analysis and the gene enrichment analysis between C1 (Fig. 3A, B, E, G), C2 (Fig. 3C, D, F, H) and the uninvolved 19 uninvolved normal tissues. Fifty-nine and 32 DDR-related genes were found to show contrasting expression between C1 and C2 group compared to uninvolved tissue, the details are shown in Table S2.

### 3.2 Identification and construction of the DDR-related key gene signature

**Fig. 4:**
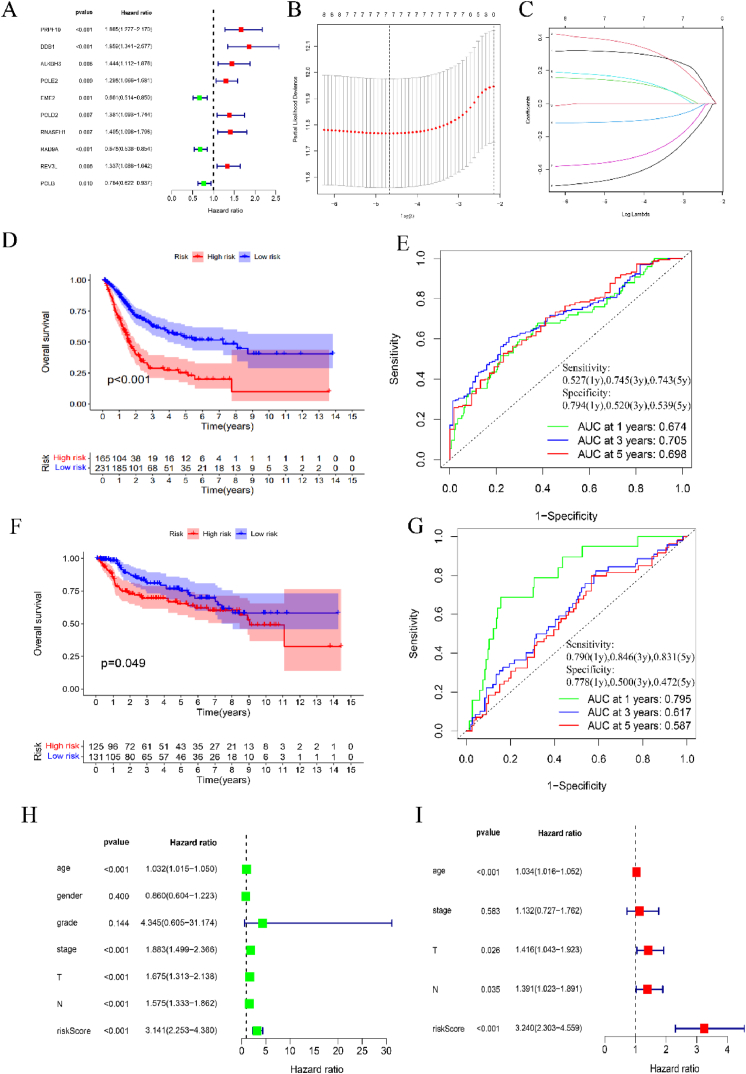
(A) Univariate Cox regression analysis of the 10 key DDR-related genes. (B, C) Construction of the LASSO Cox regression model based on 7 genes. (D, E) Kaplan–Meier plot and ROC curves for 7‐gene signature in the TCGA training cohort. (F, G) Kaplan–Meier plot and ROC curves for 7‐gene signature in the GEO validation cohort. (H, I) Univariate and multivariate Cox regression analyses on the 7-gene signatures and OS of BLCA patients in the TCGA training sets.

10 key genes, including PRPF19, DDB1, ALKBH3, POLE2, EME2, POLD2, RNASEH1, RAD9A, REV3L, POLB were screened out based on the univariate risk analysis results (Fig. 4A). LASSO Cox regression was utilized to construct a model with the minimum lambda value and enrolled 7 genes to form the final model. Riskscore = PRPF19*(0.3116) + DDB1 *(0.3758) + ALKBH3*(0.1375) + EME2*(−0.1100) + POLE2*(0.1609) + POLB*(0.3469) + RAD9A*(−0.4511) (Fig. 4B–C). As is shown in Fig. 3C, each curve with different colors in the figure represents the change track of each independent variable coefficient. The coefficients of selected features are shown by lambda parameter. The abscissa represents the value of lambda, and the ordinate represents the coefficients of the independent variable. 408 BLCA patients from the database of TCGA were enrolled for evaluate the efficacy of survival prediction of this model. The OS of high-risk patients is significantly shorter than that of low-risk patients (p < 0.001) (Fig. 4D). The ROC curves based on this model showed that OS could be effectively predicted the OS with a relatively good AUC value (Fig. 4E). To further validate the predictive efficacy of the prognosis model, we also introduced another validation cohort, which enrolled a total of 256 BLCA patients from GSE13507 and GSE31684 of GEO database. The cutoff values for validation cohort are based on the training cohort. In this different patient cohort, the prognosis model achieved a similar efficacy in predicting the OS of BLCA patients (Fig. 4F–G). The combinative analysis verified the predictive efficacy of our 7-gene DDR prognosis model for BLCA patients. Moreover, we performed the univariate and multivariate Cox regression analyses on the OS of BRCA patients in the training cohort, which suggested that the 7-gene signature could serve as an independent prognosis factor independent of other clinical risk factors (p < 0.001, HR = 3.240 (2.303–4.559) (Fig. 4H–I). In addition, the analysis of prognosis prediction based on subgrouping of different clinical parameters were also performed (Fig. S1), which indicated the extensive universality of our 7-gene model for the BLCA patients under varied clinical statuses.

### 3.3 Construction of a nomogram for OS prediction in BLCA patients

**Fig. 5:**
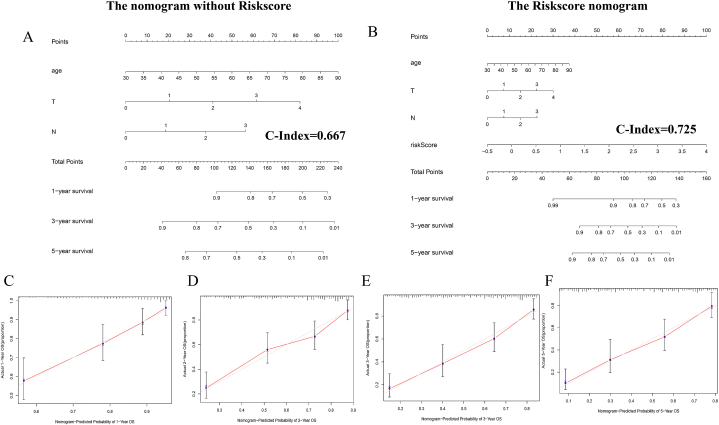
Establishment of a nomogram for OS prediction in BLCA patients. (A) The nomogram without Riskscore. (B) The nomogram combining the 7-gene signature model and age and stage to predict the OS of BLCA patients. (C–F) The plots illustrating the calibration of the nomogram measuring the consistency between the prediction and the data in the real world.

For establishing an effective and applicable method to clinically predict OS of BLCA patients, we integrated the DDR-related 7 key gene signature riskScore, age, T stage, N stage in the TCGA cohort to construct a nomogram for 1-, 3- and 5-survival prediction (Fig. 5B). Furthermore, calibration plots were drawn, which suggested a good performance played by the nomogram as an ideal model with a satisfactory predictive accuracy Fig. 5C–F). To compare the predictive efficacy and show the advantage of our 7-gene model, we also construct a Nomogram without the Riskscore (Fig. 5A). As could be seen from the result, the C-index of the nomogram without Riskscore is 0.667, which is lower than the C-index of the Riskscore nomogram (0.725).

### 3.4 Comparison of clinical parameters between different risk groups and 7 key genes expression

**Fig. 6:**
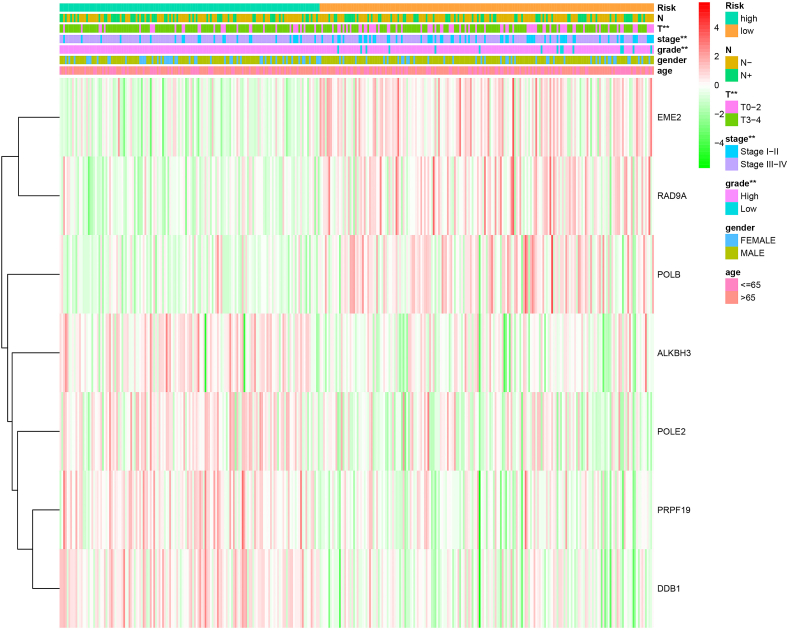
Clinicopathological features compared between the high-risk group and the low-risk group. Higher tumor grade, higher tumor stage, and T stage patients were found significantly more in the high-risk group than the low-risk group. Meanwhile, the N stage, gender and age showed no significant difference.

To further evaluate the value of our model and the 7 key genes in the clinical diagnosis of BLCA, we compared the crucial clinicopathological features of N stage, T stage, overall stage, tumor grade between the high-risk group and the low-risk group (Fig. 6). Notably, in terms of the degree of tumor malignancy represented by T stage, overall stage and tumor grade, the high-risk group demonstrated a significantly worse clinical phenotype compared with the low-risk group. By the chi-square test analysis, we found that the number of higher tumor grade, higher tumor stage, and T stage patients is significantly higher in the high-risk group than the low-risk group. Meanwhile, the N stage, gender and age showed no significant difference.

### 3.5 Gene set enrichment analysis and biological functions prediction

**Fig. 7:**
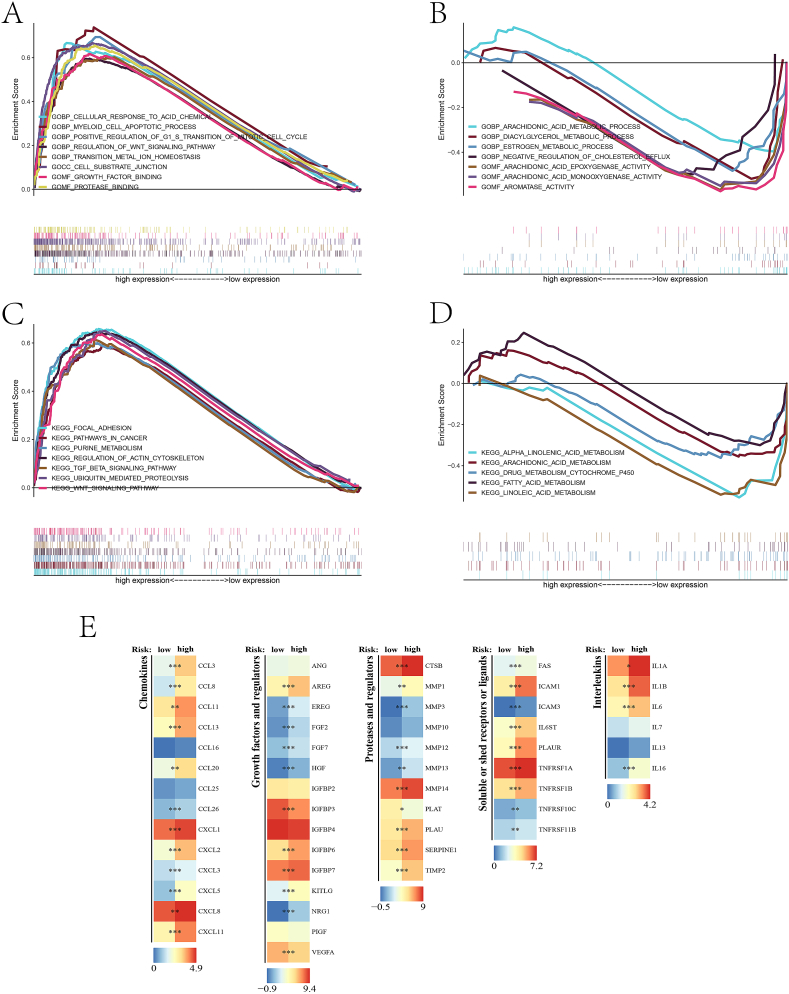
(A–D) GSEA gene set enrichment analysis. (E) Comparison of crucial small-molecular bioactive components between high-risk and low-risk groups of BLCA patients.

GSEA enrichment analysis was performed to make a deeper prediction of the enriched gene pathways differed by the DDR-related key gene signature. Top GO (Fig. 7A, B) and KEGG (Fig. 7C, D) pathways were enriched in the high-risk and low-risk groups of BLCA patients. As is shown in the figure, we can see that in the high-risk group, several crucial oncogenic pathways were predicted, including growth factor binding, positive regulation of mitotic cell cycle, TGF-beta signaling pathway and WNT signaling pathway. Also, in the low-risk group, a range of gene pathways, such as fatty acid metabolism, negative regulation of cholesterol efflux, estrogen metabolic process and so on, which mostly focused on the metabolism-related pathways. In addition, different groups of small-molecule bioactive components, such as chemokines and key receptors and ligands, were found to show a significant expression difference between high-risk and low-risk group BLCA patients (Fig. 7E).

### 3.6 Immune infiltration analysis

**Fig. 8:**
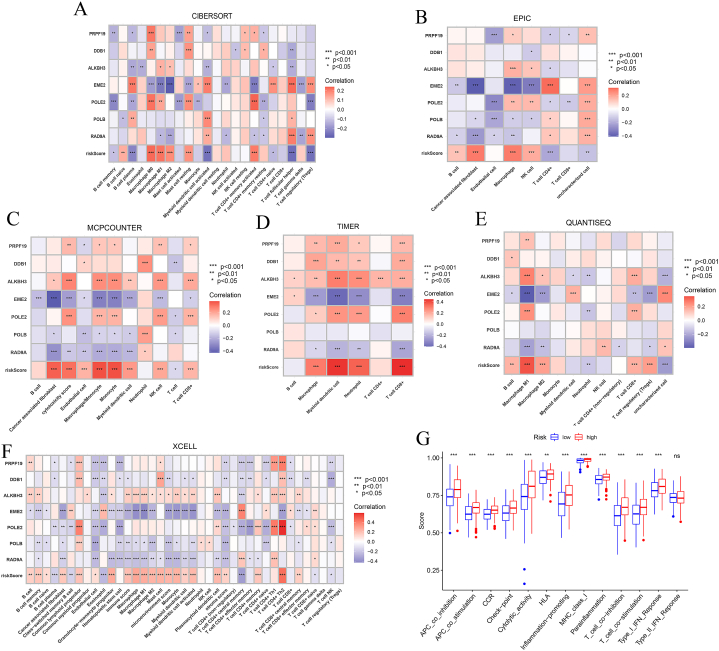
Immune infiltration analysis. (A–F) Correlation between 7 key genes and Riskscore with different immune cells with differed algorithms. (G) Comparison regarding crucial immune-regulatory processes between high-risk and low-risk groups (*p < 0.05, **p < 0.01, ***p < 0.001).

**Fig. 9:**
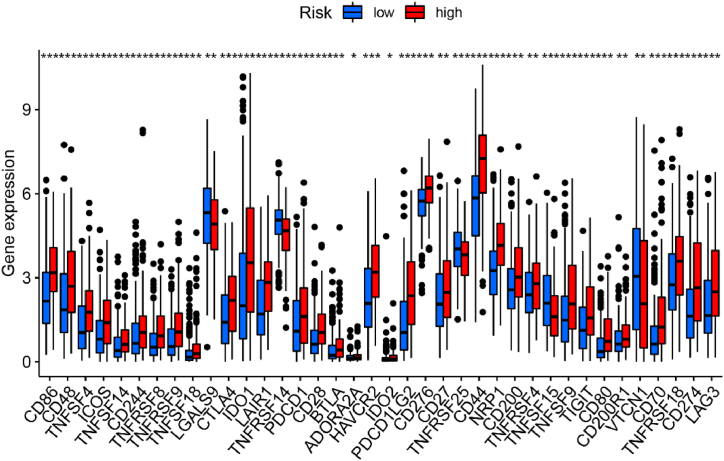
Comparison of immune-checkpoint proteins expression between high-risk and low-risk groups based on TCGA database (*p < 0.05, **p < 0.01, ***p < 0.001).

To further explore whether the DDR pathway subtyping has an impact on the immune status of BLCA microenvironment, we performed the immune scoring analysis between high-risk and low-risk groups. Significant differences in various immune cells infiltration were observed between two groups, including a range of innate immune cells and adaptive immune cells. Also, we conducted the immune correlation analysis based on the level of single gene expression across the 7 key genes (Fig. 8A–G). In these key genes, we found that multiple genes had a significant negative correlation with many crucial immune proteins, especially EME2 and RAD9A. On the contrary, the Riskscore derived from the model had a significant positive association with a range of immune proteins. In addition, we also performed an analysis on the crucial immune-related biological processes. Interestingly, the high-risk group demonstrated a significantly higher immune-scoring in almost all the immune-regulatory processes compared with the low-risk group (Fig. 8H). Recently, the expression of immune-checkpoint proteins is an important factor to evaluate the immune status and guide the administration of ICB treatment. Therefore, we also explored the difference in the expression of a range of immune-checkpoint proteins between the high-risk group and low-risk group BLCA patients (Fig. 9). Interestingly, a group of crucial immune-checkpoint proteins, such as PD-L1, PD-1, CTLA-4, IDO1, TIGIT, LAG-3 and so on, were found to be significantly up-regulated in high-risk groups compared with the low-risk groups.

### 3.7 Drug sensitivity analysis

**Fig. 10:**
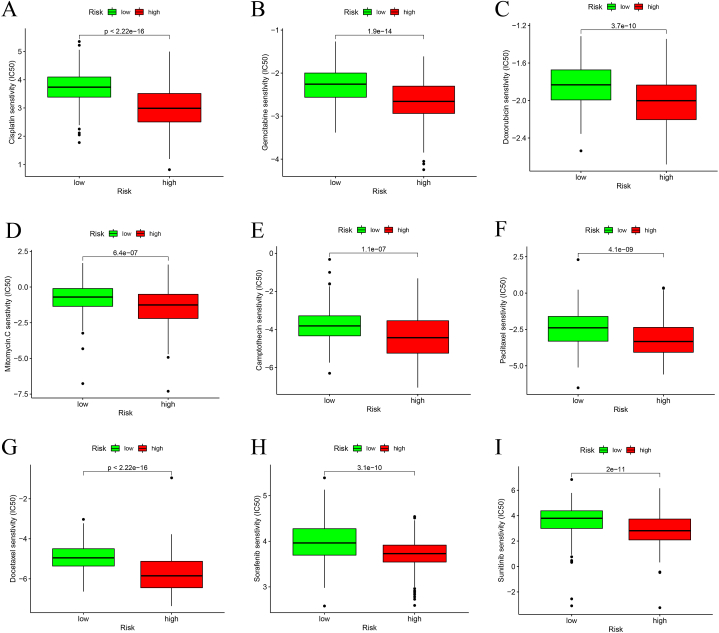
Drug sensitivity analysis based on IC50 scoring. (A) Cisplatin. (B) Gemcitabine. (C) Doxorubicin. (D) Mitomycin. (C, E) Camptothecin. (F) Paclitaxel. (G) Docetaxel. (H) Sorafenib. (I) Sunitinib.

To further determine whether our establishment of the DDR-model could guide the clinical practice on drug administration and predict the treatment efficacy for the BLCA patients, we utilized the Spearman correlation analysis of IC50 score between high-risk and low-risk groups of BLCA patients. As is shown in Fig. 10A–I, the sensitivity to 9 commonly-used chemotherapy agents were compared between low-risk and high-risk groups. Intriguingly, the results are in a great consistency to show that the BLCA patients in the high-risk group demonstrated significantly higher sensitivity to these drugs, which suggested that DDR-gene signature may participate in the formation or alteration of certain resistance to chemotherapy.

### 3.8 Immune affinity and sensitivity exploration

**Fig. 11:**
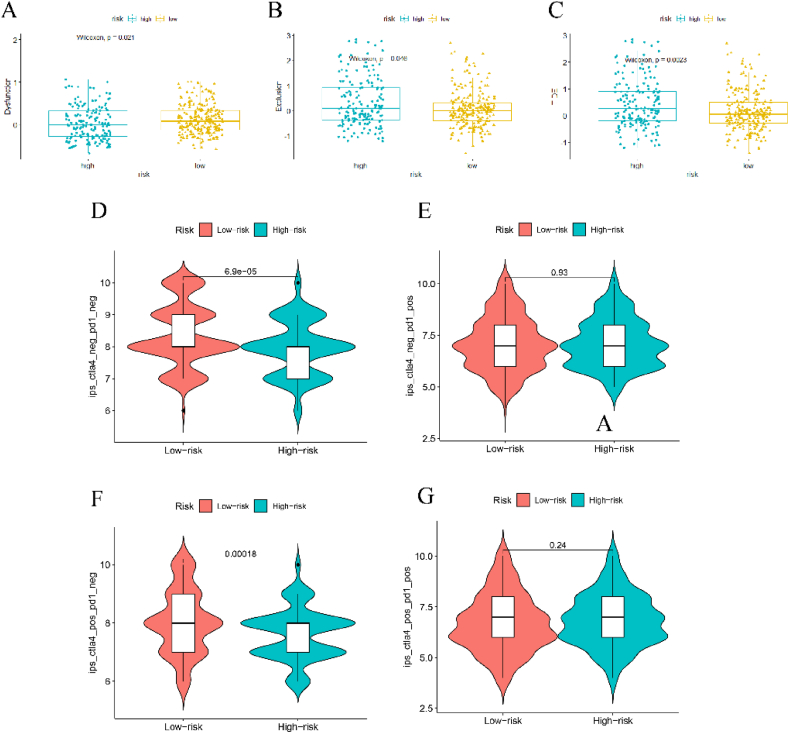
Immune affinity and sensitivity exploration. (A) TIDE scoring of immune dysfunction. (B) TIDE scoring of immune exclusion. (C) TIDE scoring of immune escape. (D–G) TCIA analysis of T cell exhaustion.

TIDE scoring algorithm is an effective approach to measuring the immune escape status in the tumor microenvironment and guide the administration of ICB therapy. Therefore, to evaluate the value of the 7-gene model in predicting the immune affinity and immunotherapy sensitivity, we performed the TIDE analysis comparing the TIDE score between the high-risk and low-risk group (Fig. 11A–C). With regards to immune dysfunction, the low-risk group showed a significantly higher score than the high-risk group, which suggested that the low-risk group exhibited a worse immune-functioning status. Nevertheless, the high-risk group displayed a significantly higher score in immune exclusion and the total TIDE score than the low-risk group, which indicated that the high-risk group is more possible to escape the immune surveillance and response poorly to ICB therapy. In addition, we performed an analysis on the immune-exhaustion status of adaptive immune T cells based on the TCIA database, with a focus on the co-expression of CTLA-4 and PD-1 (Fig. 11D–G). We found that the expression of CTLA-4^neg^PD-1^neg^T cells and CTLA-4^pos^PD-1^neg^T cells in the high-risk group is significantly higher than that in the low-risk group. While the expression of CTLA-4^neg^PD-1^pos^ and CTLA-4^pos^PD-1^pos^T cells displayed no difference between the high-risk and low-risk groups.

## 4 Discussion

BLCA is one of the most common and aggressive urological malignancies with great molecular and pathological heterogeneity [1,2]. Although early-stage BLCA could be handled with several inhibitory strategies such as TUR-BT and BCG intravesical perfusion therapy, the rate of recurrence and progression remained high [4,5]. Once the NMIBC progressed into MIBC, the possibility of occurrence of lymph node metastasis would increase sharply, which may easily develop into metastatic BLCA and the survival of patients would become extremely worse [6,7]. Recently, the emergence of ICB therapy shed light of hope on the high-stage BLCA patients, while the immune status possessed great heterogeneity between individuals, which resulted in varied response to ICB from different groups of BLCA patients [29,30]. Therefore, effective biomarkers prognosis predictors are urgently warranted for better subgrouping of BLCA patients to make precise clinical decision. Increasing evidence has indicated that the establishment of key gene signature model based on crucial biological genetic processes, such as hypoxia, immune infiltration, ferroptosis, lipid metabolism, and so on, could serve as effective tools for molecular subgrouping and prognosis prediction for multiple cancers [[31], [32], [33]]. Likewise, DNA damage repair, which is a highly-conserved biological process, has been found to be able to form prognosis prediction models based on a range of crucial related genes [34]. Here in this study, a 7-gene signature model based on DDR pathway was established by us in BLCA, which demonstrated a great accuracy in prognostic prediction and promising potential in ICB treatment guidance.

Firstly, to evaluate whether the differentially-expressed genes in DDR pathway could effectively subgroup the BLCA, two molecular subgroups of BLCA were derived based on the 232 DDR-related genes (Fig. 1A–D). Through differentially-expressed genes detection (Fig. 2B) and gene pathway enrichment analysis (Fig. 2C), we found that the two molecular subtypes exhibited significantly different characteristics, including DNA replication, nuclear division, mismatch repair, cell cycle and cellular senescence, which are all important phenotypes and biological functions closely related with the DDR process. Hence, we may conclude that the regulatory roles played by DDR-related genes possessed great heterogeneity among BLCA patients and distinct molecular subtypes could form dependent on DDR difference. In addition, within the candidate DDR-related genes, combination of a range of crucial genes may help establish a more convenient and specific prognostic model for cancer diagnosis. Recently, multiple studies utilized DDR-related genes to form a range of effective prognostic models for several cancers, such as lung cancer [35], breast cancer [36], glioma [37] and cervical carcinoma [38]. While in BLCA, a range of prognostic models have also been established. Cao et al. developed a 7-gene model based on EMT in BLCA, which demonstrated a good effect in predicting BLCA patients’ prognosis and the derived riskscore may serve as an independent risk factor for BLCA diagnosis [39]. However, this study only explored the prognostic value of the EMT-signature in BLCA, and whether this tool could guide the ICB or chemotherapy selection is still unclear. Moreover, the biological mechanism remained unknown, which limited the further clinical use of this model. Zhou et al. also established a 11-gene signature based on autophagy for prognosis prediction in BLCA, which exhibited an even better efficacy in survival prediction. Nevertheless, a same defect still existed in this study that the inner mechanism explanation lacked [40]. More recently, ferroptosis has been identified as a novel iron-dependent cell death regulatory mechanism and has also been applied as a prognostic modality in BLCA. Notably, the established ferroptosis-based riskscore was found to be associated with the expression of a range of immune-checkpoint proteins in the tumor microenvironment of BLCA [41]. This study suggested that the gene signature may not only serve as a prognosis predictive tool, but also could represent the inner immune status and mark the suitability of BLCA patients for ICB therapy. As mentioned above, DDR is a pivotal modulator in regulating the progression and therapeutic response in cancer. More importantly, DDR pathways are also found to be tightly correlated with the immune activity, which may be utilized as genomic biomarkers for cancer immunotherapy. Qing et al. explored the double-sword function of DDR in the cancer immunotherapy and found that DDR pathway mutations as well as their derived up-regulated neoantigens could determine diverse immune phenotypes [42]. Likewise, another study has also identified the co-mutations in DDR system could effectively predict the outcomes of lung cancer and melanoma patients in response to ICB therapy [43]. For BLCA, the main risk factors include smoking and exposure to deleterious chemicals, which are all powerful driving force for induction of DNA damage [44]. Thus, we may naturally understand the great biological significance of DDR system in BLCA. Correspondingly, multiple studies have revealed the important regulatory roles played by DDR in BLCA. In a randomized controlled trial, the polymorphism of DDR-related genes has been found be associated with the progression of BLCA, especially muscle-invasiveness [45]. Notably, under the treatment of platinum-based chemotherapy, patients with DDR-related genetic alterations have a significantly better prognosis compared with the controls [46,47], which indicated that DDR-related information may assist in the patient selection for multiple therapeutics, including ICB therapy. Nevertheless, whether DDR-related genes could help establish an applicable and effective prognosis prediction model and guide in selection of different therapeutics is still unknown.

Based on the DDR-subgrouping of molecular subtypes in BLCA, we screened out 7 key DDR-related genes, which are all significantly-associated risk factors for BLCA prognosis, to form the final gene signature model. As shown in Fig. 4D–F, the model could effectively predict the overall survival of BLCA patients with a high accuracy in two independent datasets. And the riskscore could serve as an independent risk factor for BLCA. The relatively low number of genes provided with the convenience in developing the rapid detection kit for clinical practice to BLCA. GSEA enrichment analysis enabled us to further identified the biological functions differed by DDR features. As we can see from Fig. 7A and C, several gene pathways are significantly upregulated in the high-risk groups, including some classical oncological pathways, such as WNT signaling pathway, growth factor binding and pathway in cancer. Hyperactivation of WNT signaling pathways has been verified as a major engine force for immune escape promotion and therapeutic resistance, which is mainly dependent on enhancement of DDR [48,49]. Oxidative stress-mediated WNT pathway is a potential therapeutic target for optimizing cancer therapy via regulating genomic integrity [50]. In addition, a range of metabolic pathways have also been found to be significantly altered between the high-risk and low-risk group, including purine, fatty-acid, α linolenic-acid and arachidonic-acid metabolism. The synthesis of fatty-acid through fatty-acid synthase could up-regulate DDR and therefore induce cancer cell survival and therapeutic resistance [51]. In turn, specific DDR proteins could also modulate lipid metabolism in tumor cells [52]. In all, the interactive work between cellular metabolism and DDR is an important regulator of biological processes, which may be a valuable target for novel therapeutics design. The difference in the expression of multiple cytokines, soluble ligands and growth regulators between the high-risk and low-risk group further indicate the variation of biological phenotypes differed by DDR features (Fig. 7E).

The immune infiltration analysis showed us that the higher riskscore is positively correlated with the activation of both innate and adaptive immunity. As can be seen in Fig. 8, the expression of macrophage has a significantly-positive association with the riskscore across all the algorithms. Furthermore, the immune function analysis indicated that a range of immune pathways mainly concerning antigen-presentation processes, including APC co-stimulation, HLA and MHC-I. Alterations or deficiency in the DDR system could cause increased accumulation of genetic mutations and chromosomal rearrangement, which lead to genomic instability and may promote tumor progression [53]. Nevertheless, this process could also generate a series of neoantigens [54,55] and up-regulate immune-checkpoint proteins, such as PD-L1 [56,57], which may exert immune-activating functions and could serve as a synergic functioning target in combination with ICB agents. In BLCA, Zeng et al. found the macrophage infiltration, especially M1 subtype is an effective biomarker for predicting ICB response and determining immune characteristics in the tumor microenvironment. Notably, M1 expression is positively associated with T cell immunity and PD-L1 expression and negatively correlated with immune exclusion [58]. Under the premise that abnormal regulation of DDR system and increased accumulation of neoantigens, macrophages could also exert their antigen-presentation capabilities, which would also active adaptive T cell anticancer immunity [59,60]. However, the cross-presentation of macrophages could also induce immune tolerance. In consistency with the results of the immune infiltration analysis, the immune-checkpoint protein analysis indicated that the high-risk group exhibited a significantly higher expression of a range of immune-checkpoint proteins, including PD-L1, PD-1 and CTLA-4 (Fig. 9). Higher PD-L1 expression in tumor commonly represented an immune-sensitive status, which confers the patients with better response to ICB therapy [61,62]. On the other hand, the over-expression of immune-checkpoint like PD-1 on CD8^+^ T cell represented an immune-exhausted state, which could be recovered by anti-PD-1 ICB therapy and reinvigorate the anti-tumor immunity [63,64]. In our TIDE scoring analysis, we found that the overall immune escape phenotype and immune exclusion in high-risk group is significantly better than the low-risk group, which indicated that our model-derived high-risk group of BLCA patients may be an ideal subgrouping selection for better responders to ICB therapy. In addition, the PD-1-negative T cell expression is significantly lower in high-risk group than that in the low-risk group (Fig. 11D and F), which further verified the great potential of immune reactivation in high-risk group patients of BLCA.

In addition to the ICB therapy, considering that the standard therapeutics for BLCA are still centered on chemotherapy and molecule-targeted therapy, we also performed a drug sensitivity analysis on a series of commonly-used drugs for BLCA based on the DDR model grouping. Surprisingly, we found that the high-risk group patients displayed a significantly better sensitivity to all the 9 analyzed agents (Fig. 10). Cisplatin is the first metal-based antitumor chemotherapeutic agent, which is still the standard first-line chemotherapy regimen for BLCA. The main anticancer mechanism involves its cytotoxic interaction with the DNA bases and the induction of cellular apoptosis [65,66]. The core of the tumor-killing capability of cisplatin is dependent on its promotion of DNA damage, which could be resisted innately or adaptively by the up-regulation of DDR processes [67]. Wang et al. found that the DDR key gene BRCA1 could be inhibited by XPC inhibition in BLCA, which could lead to increased chromosomal instability and maintaining cisplatin-induced DNA damage [68]. Moreover, DDR-related genes have been found to be able to effectively predict disease-free survival for muscle-invasive BLCA [69]. The same effect has also been found in Doxorubicin, Mitomycin and Camptothecin, which are mainly dependent on the DNA-cytotoxicity. From these results we may conclude that DDR itself may be a favorable factor for maintaining genomic stability in a homeostasis state. However, under the treatment of DNA-cytotoxicity dependent chemotherapy, the up-regulation of DDR may be an unfavorable factor for forming drug resistance. In all, our 7-gene model based on DDR gene signature could also serve as an effective tool for BLCA patient selection of different therapeutics.

## Author contribution statement

Hongqian Guo; Rong Yang: Conceived and designed the experiments.

Tianhang Li; Ning Jiang; Yuhao Bai: Performed the experiments; Analyzed and interpreted the data; Wrote the paper.

Tianyao Liu; Zihan Zhao; Xinyan Xu: Contributed reagents, materials, analysis tools or data.

Yulin Zhang; Fayun Wei; Rui Sun: Performed the experiments; Wrote the paper.

Siyang Liu; Jiazheng Li: Analyzed and interpreted the data; Wrote the paper.

## Funding statement

This work was supported by the 10.13039/501100001809National Natural Science Foundation of China (82172691 and 81772710) and Nanjing Science and 10.13039/100006180Technology Development Key Project (YKK19011).

## Data availability statement

The differentiated-expressed gene data and clinical information were all extracted from the TCGA and GEO database, where all the data were up-loaded and shared voluntarily and could be downloaded and analyzed freely.

## Declaration of interest's statement

The authors declare that they have no known competing financial interests or personal relationships that could have appeared to influence the work reported in this paper.

## Abbreviations

BLCA: Bladder cancer
NMIBC: Non-muscle invasive bladder cancer
TURBT: Transurethral resection of bladder tumor
ICB: Immune-checkpoint blockade
DDR: DNA damage repair

## Appendix A Supplementary data

Multimedia component 2 Multimedia component 2

figs1 figs1
