# Supplementary material for: Prediction of immune infiltration and prognosis for patients with urothelial bladder cancer based on the DNA damage repair-related genes signature
Source: Heliyon. 2023 Feb 13;9(3):e13661. doi: 10.1016/j.heliyon.2023.e13661 (PMC9976330; doi:10.1016/j.heliyon.2023.e13661)
Supplement: Multimedia component 1 [file mmc1.docx]

| **All the candidate DDR-related Genes** |
| --- |
| RAD23A |
| RAD17 |
| RMI2 |
| RAD54L |
| UBE2N |
| MED1 |
| POLG |
| RECQL |
| ENDOV |
| PRPF19 |
| APEX2 |
| BRCA2 |
| RPA3 |
| RNASEH2B |
| RAD18 |
| ATRIP |
| ERCC8 |
| PRKDC |
| BRIP1 |
| DDB1 |
| ERCC5 |
| BIVM-ERCC5 |
| POLQ |
| CHEK1 |
| XPA |
| NUDT1 |
| FANCG |
| RFC5 |
| ALKBH3 |
| POLA2 |
| RDM1 |
| XRCC1 |
| CCNL1 |
| FANCA |
| LIG1 |
| MLH1 |
| CHEK2 |
| PARP4 |
| EME1 |
| RPA1 |
| ERCC3 |
| FEN1 |
| DNA2 |
| RAD1 |
| XRCC6 |
| TREX1 |
| POLE |
| LIG4 |
| FAN1 |
| OGG1 |
| GEN1 |
| SLX1B |
| POLE2 |
| POLN |
| RFC3 |
| MPG |
| XRCC5 |
| HES1 |
| HMGB1 |
| RBBP8 |
| MRE11 |
| MBD4 |
| POLD4 |
| XRCC2 |
| RNASEH2A |
| MUS81 |
| BACH1 |
| RFC1 |
| UBE2A |
| RNF8 |
| TELO2 |
| EME2 |
| TDP2 |
| PRIM1 |
| RAD52 |
| MCM6 |
| MCM7 |
| POLD2 |
| ECD |
| MSH4 |
| POLD1 |
| CENPX |
| FANCF |
| TOP3A |
| NEIL3 |
| RECQL5 |
| TOP3B |
| REV1 |
| RAD51C |
| BLM |
| TREX2 |
| RNF4 |
| NEIL1 |
| TDG |
| FANCI |
| RIF1 |
| PER1 |
| RFC4 |
| RAD54B |
| RPA2 |
| WDR48 |
| DCLRE1C |
| POLA1 |
| SMUG1 |
| POLL |
| EXO1 |
| RECQL4 |
| CDK7 |
| MAD2L2 |
| H2AFX |
| ERCC6 |
| JUNB |
| FANCB |
| POLE4 |
| HLTF |
| RAD50 |
| TOPBP1 |
| DNTT |
| RRM2B |
| BRCA1 |
| UBE2B |
| DCLRE1A |
| APLF |
| LIG3 |
| MUTYH |
| FANCD2 |
| MGMT |
| NHEJ1 |
| ATR |
| APTX |
| MLH3 |
| DUT |
| PMS2 |
| XPC |
| ALKBH2 |
| GTF2H5 |
| NTHL1 |
| PARP3 |
| CHAF1A |
| RBX1 |
| TDP1 |
| RAD51 |
| SHPRH |
| NBN |
| SPRTN |
| POLD3 |
| CLK2 |
| WRN |
| RAD51D |
| HUS1 |
| CUL4B |
| XAB2 |
| PMS2CL |
| APEX1 |
| SSBP1 |
| TP53BP1 |
| MSH5 |
| FANCM |
| GTF2H1 |
| NEIL2 |
| MSH6 |
| GTF2H2 |
| GTF2H2C |
| CCNH |
| SETMAR |
| SLX1A |
| RAD23B |
| SPO11 |
| RNASEH1 |
| CETN2 |
| FANCC |
| UBE2T |
| RPA4 |
| MNAT1 |
| HAP1 |
| ERCC1 |
| RNH1 |
| UVSSA |
| RAD9A |
| XRCC4 |
| RNASEH2C |
| SYCP3 |
| FANCE |
| MSH3 |
| PMS1 |
| FAAP24 |
| REV3L |
| RAD51B |
| POLM |
| MMS19 |
| DDB2 |
| ERCC2 |
| XRCC3 |
| POLK |
| MCM2 |
| HELQ |
| MSH2 |
| DCLRE1B |
| ATM |
| POLH |
| RFC2 |
| USP1 |
| RNF168 |
| FANCL |
| SEM1 |
| CUL4A |
| PCNA |
| MCM4 |
| PRIM2 |
| POLI |
| GTF2H3 |
| MDC1 |
| PALB2 |
| POLB |
| PNKP |
| MCM3 |
| FAH |
| RMI1 |
| MCM5 |
| ERCC4 |
| DMC1 |
| TP53 |
| UNG |
| GTF2H4 |
| PARP1 |
| SLX4 |
| CENPS |
| UBE2V2 |
| FAAP20 |
| FAAP100 |
| POLE3 |
| PARP2 |
