# Supplementary material for: Prediction of immune infiltration and prognosis for patients with urothelial bladder cancer based on the DNA damage repair-related genes signature
Source: Heliyon. 2023 Feb 13;9(3):e13661. doi: 10.1016/j.heliyon.2023.e13661 (PMC9976330; doi:10.1016/j.heliyon.2023.e13661)
Supplement: Multimedia component 2 [file mmc2.docx]

| **Significant different DDR-related genes compared with uninvolved tissues** | |
| --- | --- |
| **C1** | **C2** |
| RMI2 | RMI2 |
| RAD54L | RAD54L |
| BRCA2 | POLQ |
| RPA3 | NUDT1 |
| RAD18 | RDM1 |
| BRIP1 | FANCA |
| POLQ | LIG1 |
| CHEK1 | EME1 |
| NUDT1 | FEN1 |
| FANCG | HES1 |
| RFC5 | XRCC2 |
| POLA2 | RNASEH2A |
| RDM1 | POLD1 |
| FANCA | CENPX |
| LIG1 | BLM |
| EME1 | FANCI |
| FEN1 | PER1 |
| DNA2 | SMUG1 |
| POLE | EXO1 |
| GEN1 | RECQL4 |
| POLE2 | H2AFX |
| RFC3 | JUNB |
| XRCC2 | FANCD2 |
| RNASEH2A | RAD51 |
| PRIM1 | CLK2 |
| MCM6 | UBE2T |
| MCM7 | RAD9A |
| POLD1 | FAAP24 |
| CENPX | REV3L |
| NEIL3 | MCM2 |
| BLM | SEM1 |
| FANCI | PNKP |
| PER1 |  |
| RFC4 |  |
| EXO1 |  |
| RECQL4 |  |
| MAD2L2 |  |
| H2AFX |  |
| JUNB |  |
| FANCB |  |
| TOPBP1 |  |
| BRCA1 |  |
| FANCD2 |  |
| CHAF1A |  |
| RAD51 |  |
| UBE2T |  |
| FANCE |  |
| FAAP24 |  |
| XRCC3 |  |
| MCM2 |  |
| MSH2 |  |
| DCLRE1B |  |
| RFC2 |  |
| PCNA |  |
| MCM4 |  |
| PALB2 |  |
| RMI1 |  |
| MCM5 |  |
| UNG |  |
