# Supplementary figures and images for: Prediction of immune infiltration and prognosis for patients with urothelial bladder cancer based on the DNA damage repair-related genes signature
Source: Heliyon. 2023 Feb 13;9(3):e13661. doi: 10.1016/j.heliyon.2023.e13661 (PMC9976330; doi:10.1016/j.heliyon.2023.e13661)

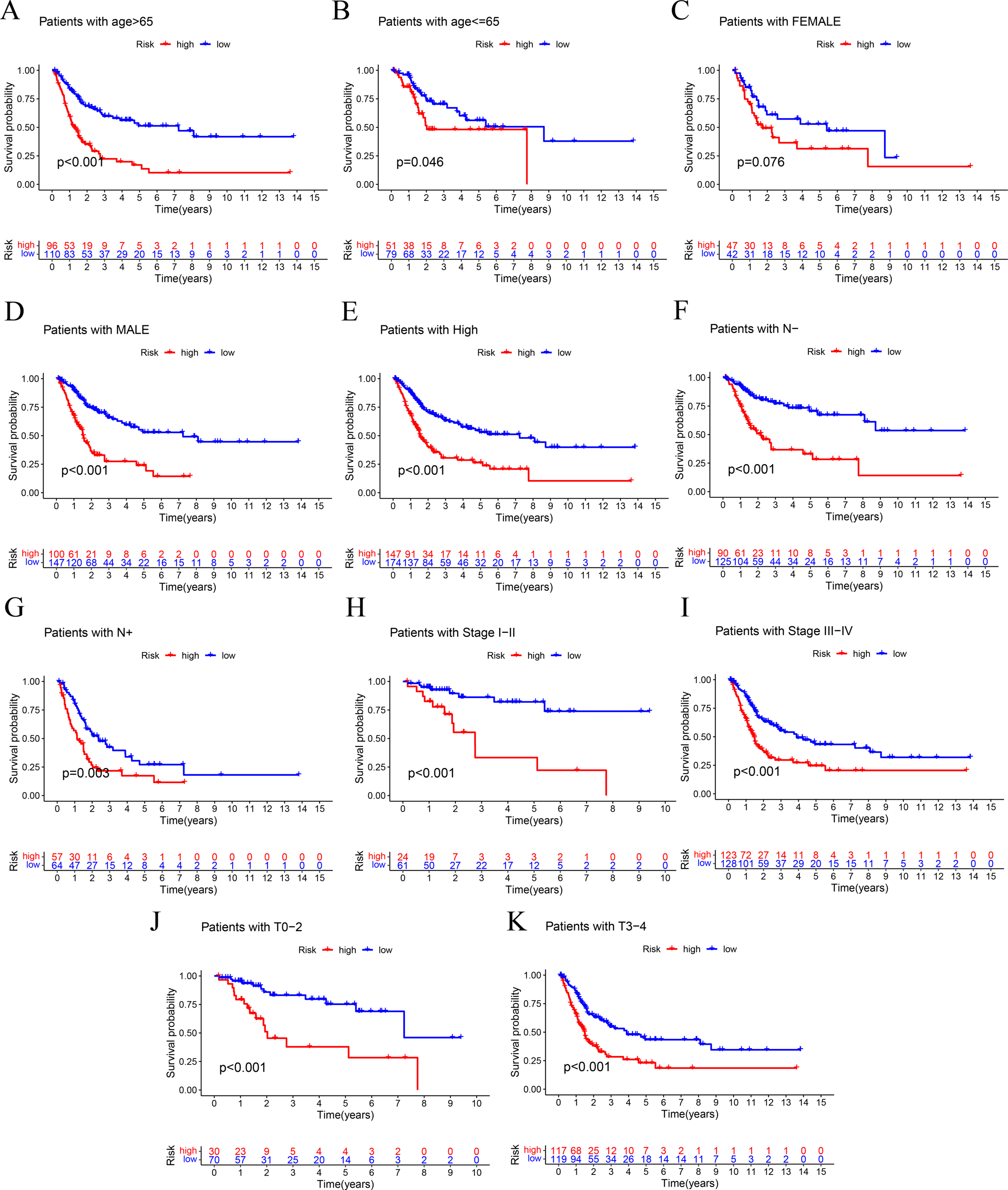

Supplement: figs1 [file mmcfigs1.jpg]
